# Optimizing cost-efficiency in mean exposure assessment - cost functions reconsidered

**DOI:** 10.1186/1471-2288-11-76

**Published:** 2011-05-21

**Authors:** Svend Erik Mathiassen, Kristian Bolin

**Affiliations:** 1 Centre for Musculoskeletal Research, Department of Occupational and Public Health Sciences, University of Gävle, Sweden; 2 Department of Economics, Lund University, Lund, Sweden

## Abstract

**Background:**

Reliable exposure data is a vital concern in medical epidemiology and intervention studies. The present study addresses the needs of the medical researcher to spend monetary resources devoted to exposure assessment with an optimal cost-efficiency, i.e. obtain the best possible statistical performance at a specified budget. A few previous studies have suggested mathematical optimization procedures based on very simple cost models; this study extends the methodology to cover even non-linear cost scenarios.

**Methods:**

Statistical performance, i.e. efficiency, was assessed in terms of the precision of an exposure mean value, as determined in a hierarchical, nested measurement model with three stages. Total costs were assessed using a corresponding three-stage cost model, allowing costs at each stage to vary non-linearly with the number of measurements according to a power function. Using these models, procedures for identifying the optimally cost-efficient allocation of measurements under a constrained budget were developed, and applied on 225 scenarios combining different sizes of unit costs, cost function exponents, and exposure variance components.

**Results:**

Explicit mathematical rules for identifying optimal allocation could be developed when cost functions were linear, while non-linear cost functions implied that parts of or the entire optimization procedure had to be carried out using numerical methods.

For many of the 225 scenarios, the optimal strategy consisted in measuring on only one occasion from each of as many subjects as allowed by the budget. Significant deviations from this principle occurred if costs for recruiting subjects were large compared to costs for setting up measurement occasions, and, at the same time, the between-subjects to within-subject variance ratio was small. In these cases, non-linearities had a profound influence on the optimal allocation and on the eventual size of the exposure data set.

**Conclusions:**

The analysis procedures developed in the present study can be used for informed design of exposure assessment strategies, provided that data are available on exposure variability and the costs of collecting and processing data. The present shortage of empirical evidence on costs and appropriate cost functions however impedes general conclusions on optimal exposure measurement strategies in different epidemiologic scenarios.

## Background

Reliable exposure assessment is a vital concern in medical epidemiology and intervention research. In occupational as well as public health studies, exposure is often monitored using equipment that allows data to be collected at a high resolution for long periods and on repeated occasions (e.g. [1-4]). A considerable emphasis has been put on developing and applying methods for analyzing sources of exposure variability in such data, in terms of so-called variance components [5-8]. As an example, variance components pertaining to, e.g. companies, occupations, subjects, days within subjects, and exposure samples within days have been determined for a large number of airborne, dermal, and biomechanical exposures in working life (e.g. [2,3,9-15]). These variance components have been utilized as a remedy for identifying targets for surveillance, intervention and prevention [6,16,17], as well as for designing effective exposure assessment strategies producing information at a desired level of precision. While an extensive literature deals with the consequences of random exposure variability to bias and precision in exposure-outcome relationships [18-22], some attention has also been paid to the use of variance components for estimating sampling needs in studies examining compliance with exposure limits [6], and in studies comparing groups [12] or conditions [13] as in an intervention scenario. In the latter case, the requirement for reliable exposure data can be expressed as a need to obtain estimates of the mean exposure of individuals or groups with a sufficient precision to arrive at a confidence interval of acceptable size, or secure an acceptable statistical power in a specified hypothesis test. Generalized formulae are available for estimating statistical efficiency, i.e. the relationship between the precision of a mean exposure estimate, on the one hand, and, on the other, the size of relevant variance components, and the number of measurements at the corresponding sampling stages [23,24]. The most frequently applied measurement model is hierarchical and random with two or three nested stages, for instance subjects and days within subjects [2,25,26]; subjects, days within subjects and samples within days [12,27]; or groups, subjects within groups, and days within subjects [28]. A few attempts have been made to apply more complicated models, e.g. including crossed (non-nested) components related to the distribution of measurement days among subjects [29] or associated with methodological variance [11]. Also, mixed models including fixed determinants of exposure in addition to random effects are in increasing use [13,30-33].

Some studies have been devoted particularly to understanding the effects on the precision of an estimated group mean exposure of allocating measurement efforts in different ways between and within subjects [12], between occupational recordings and data processing [11], or across time within a measurement day [34,35]. This had led to a number of principles for statistically efficient exposure assessment, i.e. measurement strategies that perform well at a specified investment of measurement resources, or, equivalently, yield a specified performance with comparatively small measurement efforts [12,34]. As one trivial conclusion, more data generally leads to better statistical performance, and furthermore, efficiency increases if measurements are allocated to higher sampling stages in the hierarchical model [23].

At the same time, more measurements inevitably imply larger monetary costs. While budget constraints are the pragmatic reality in most exposure assessments, surprisingly few studies have addressed the issue of how to design a measurement strategy so as to give the best possible statistical efficiency at the available *monetary *resources [36]. This endeavor is not equivalent to addressing statistical efficiency *per se*, as introduced above, since measurements at different stages may entail different costs. For instance, increasing the number of groups may be considerably more expensive than collecting data from more subjects in an existing group; and the process of identifying and approaching a new subject may be more expensive than achieving more measurements from a subject already in the sample population. Also, different measurement instruments yielding the same exposure variables may imply different costs, in particular if the risk of measurement failures is acknowledged [37]. Of the limited literature devoted to efficiency *and *cost in data collection, some studies compare a selection of measurement strategies in order to identify the one superior in cost-efficiency [38-41]. A few studies take on the more challenging task of determining the *optimally *cost-efficient strategy at a certain budget, on the basis of specified costs for collecting data at different stages, and specified sizes of the corresponding variance components. The general significance of examining cost-efficiency in data collection is illustrated by previous studies appearing in a variety of research areas, including occupational hygiene [38], environmental medicine [39,42,43], clinical chemistry [44], and nutrition [45].

**Figure 1:**
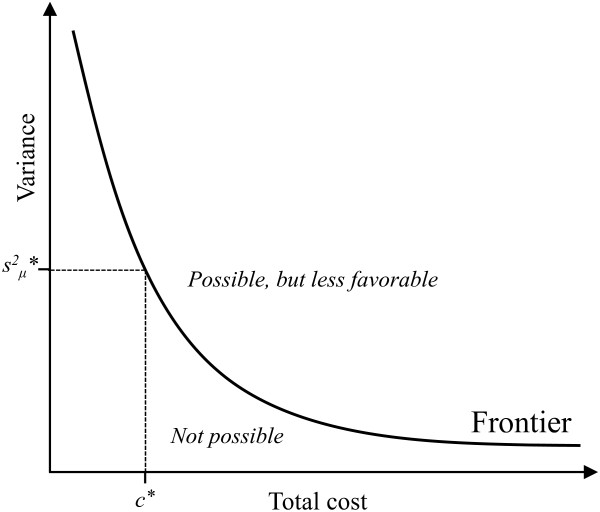
**The notion of optimal cost-efficiency**. The horizontal axis illustrates the total cost associated with an exposure measurement strategy, and the vertical axis shows the variance of the resulting mean exposure. The frontier curve illustrates the minimal obtainable variance at each level of spending, i.e. the best possible statistical performance, e.g. s^2^_μ_*, at a particular total cost, e.g. c*. Strategies *above *the frontier are, in principle, possible, but do not yield an optimal performance. No strategies occur *below *the frontier.

Basically, optimization in the case of exposure assessment strives to identify data collection strategies at the frontier of possible relationships between cost and statistical efficiency (figure 1).

Previous optimization studies have addressed hierarchical models with two [45-47] or three [43,44,47] stages, as well as the optimal allocation of measurements between two alternative yet correlated instruments for data collection [42,48,49]. All these studies have, however, assumed that the price of one measurement unit at each stage is constant, implying that costs increase in a linear fashion at that stage, proportionally to the number of samples. Only in an appendix of the paper by Duan and Mage [42], an empirical example appears of the quite likely case that costs may vary with the number of measurements; for instance that subjects recruited late in a study may require more time for being persuaded, and thus entail larger labor costs, than subjects signing up immediately. Also, in his textbook on sampling strategies, Cochran [47] reports some non-linear cost functions in other areas of data collection, and additional examples appear in Groves [50]. In addition, the cited cost-efficiency studies do not, in general, consider whether the identified optimal strategies are feasible under the constraints dictated by a specified, yet limited budget.

Thus, the present paper is devoted to deriving methods for optimizing exposure assessment strategies, in terms of offering the best possible trade-off between total costs and statistical efficiency. In contrast to previous literature, this study explores optimal cost-efficiency even when cost functions are not linear and budget constraints apply, and the study also identifies alternative optimization procedures in those cases where analytical closed-form solutions cannot be developed.

First, the paper presents a general theoretical model of cost and efficiency when assessing exposure mean values in occupational groups, including some theoretical results based on that model. Then, the general model is simplified, and procedures are derived for identifying optimally cost-efficient exposure assessment strategies, depending on the shapes of cost functions. These results are illustrated by numerical examples. A general discussion on the representativeness and sensitivity of the suggested optimization procedures concludes the paper.

## Methods

### A framework for cost-efficient exposure assessment

Exploring cost-efficiency at an ordinal level only requires a specification of the properties of the mathematical function associating each exposure assessment strategy with its stated statistical objective. If, however, the goal of the cost-efficiency analysis is to compare or optimize strategies in explicit, quantitative terms, specific functional forms need be identified that parameterize objectives and costs. This is a necessary requirement when aiming at the (occasionally more than one) strategy that maximizes efficiency among the large selection of possible assessment strategies entailing a particular cost.

Thus, three major issues must be considered as part of a quantitative analysis of cost-efficient resource consumption: (1) why resources are used, i.e. the *objective *of collecting data, (2) how much resources are required to fulfil the objective, expressed in terms of *unit-costs*, and (3) whether the intended strategy for resource consumption is *feasible*. When examining cost-efficient assessments of group mean exposure we thus need to know (1) the relationship between the group mean and the assessment strategy, as reflected by what is usually referred to as the *objective function*, (2) the amount of monetary resources required to realise a particular assessment strategy, expressed by the *cost function*, and (3) the amount of monetary resources at our disposal, as reflected by the *budget constraint*.

### The objective function - precision of the mean

For a hierarchical three-stage balanced data set (subjects, occasions within subject, samples within occasion), the group mean exposure, *μ*, can be estimated using a "mean of means" approach [23] as:

Where *x*_*k*(*ij*) _is an individual exposure sample, collected from subject *i *on occasion *j*; *n*_*s *_is the number of subjects included in the data set; *n*_*d *_is the number of distinct measurement occasions, for instance days, per subject; and *n*_*q *_is the number of samples, or quanta, per measurement occasion. Accordingly, averaging is made across quanta within each occasion, then across occasions within each subject, and finally across subjects.

A general formula for determining the variance of this group mean exposure estimate, , has been proposed and applied by several authors [12,23,44,47]. This objective function takes the form:(1)

, , and  are the variances between subjects, between measurement occasions within each subject, and between quanta within occasions, respectively. The size of a quantum can be defined as convenient, and previous studies have used quanta of, for instance, one minute [34,51], one work cycle [11,13,52,53], several consecutive work cycles [12,54], and one hour [55]. Thus, equation (1) gives an estimate of the precision of a group mean exposure resulting from a particular measurement strategy in terms of subjects, occasions and quanta, in a setting with known components of exposure variability.

### The cost function

While all cost functions suggested in the literature have been linear, the cost associated with collecting *n*_*q*_quanta on each of *n*_*d *_occasions for each of *n*_*s *_subjects can be assessed even in a non-linear case, provided that information is available on the "capability" to recruit subjects, that is, the amount of resources needed for recruiting any specific number of subjects, and the equivalent capabilities for setting up measurement occasions within each subject and collecting quanta within each occasion.

Assume first that these three capabilities are all homogeneous of degree *k*, in the sense that if all resources are multiplied by a certain factor, *x *(*x *> 1), output will increase by *x*^*k*^. This is a common assumption in economics addressing non-linear production capabilities. For example, if *k *= 1 and resources allocated to the process of recruiting subjects are doubled, then the number of subjects recruited will also double; this is simple proportional linearity. In the case of *k *= 0.5, doubled recruitment resources would lead to an increase in the number of recruited subjects by a factor . Assume further that the resources needed for setting up *n*_*d *_measurement occasions, each containing *n*_*q*_quanta, do not depend on the subject from whom data are collected, and the resources needed to collect *n*_*q *_quanta on a particular measurement occasion for a particular subject are independent of occasion and subject.

The first of these two assumed capability properties allows cost functions for recruiting subjects, *c*_*s*_, setting up measurement occasions within each subject, *c*_*d*_, and collecting measurement quanta within each occasion, *c*_*q*_, to be expressed as: ; ; and ,

where the *π*-values are the costs for obtaining one measurement unit at each stage of data collection, so-called unit costs, and *α, β *and *γ *are parameters, all larger than 0, describing the shape of a power relationship between the number of measurement units and costs.

The relationship between the value(s) of *π *and the exponents *α, β *and *γ *can be illustrated by examining the cost functions. If, for instance, *α *= 1, the cost of recruiting *n*_*s *_subjects is *c*_*s*_(*n*_*s*_) = *π*_*s *_⋅*n*_*s*_, i.e. the cost increases in direct proportion to the number of subjects. In this case, *π*_*s *_is the one-unit cost (*c*_*s *_(1) = *π*_*s*_), as well as the marginal cost of recruiting any additional subject (∂*c*_*s*_/∂*n*_*s *_= *π*_s_). If α ≠ 1, *π*_s _is still the one-unit cost, but the marginal cost is now . Thus, if *α *> 1, the marginal cost of including an additional subject increases with the number of subjects, while it decreases when 0 <*α *< 1.

The second capability property assumed above implies that the total cost of collecting a data set including *n*_*s *_subjects each observed for *n*_*d *_occasions, each containing *n*_*q *_quanta can be stated as *c*_*s *_(*n*_*s*_) + *n*_*s *_*c*_*d *_(*n*_*d*_) + *n*_*s *_*n*_*d *_*c*_*q *_(*n*_*q*_), which equals:(2)

This cost function presents a generalisation of previously suggested linear cost functions [43,44,46] by permitting both linear and non-linear relationships between the sample size at different stages of data collection and the cost of obtaining data. With (*α, β, γ*) = (1,1,1), equation (2) takes the customary linear form used in previous studies. Notably, equation (2) only expresses the variable costs associated with measurement; possible fixed costs, which do not depend on the number of samples, need to be added to give the total cost of collecting the data set, but will not affect the optimization procedures developed below [41,43].

### The general optimization problem

If a data collection is allowed to consume a total budget *R *(after possible reduction by fixed costs), combinations of *n*_*s*_, *n*_*d *_and *n*_*q *_that optimize the output, i.e. minimize the resulting variance of the estimated mean exposure, can be retrieved by solving the following optimization problem:

with respect to *n*_*s*_*, n*_*d*_*, n*_*q*_; subject to the constraint:

Due to the non-linear property of this three-variable equation system, explicit solutions for optimization can be derived only in exceptional cases. Moreover, solutions to a three-variable problem are difficult to illustrate graphically. Therefore, the following analysis will be limited to cases in which the number of quanta, *n*_*q*_, within each measurement occasion is not a choice variable. This situation occurs for instance when exposure is assessed for complete days, or when the within-day schedule of data sampling cannot or should not be manipulated for reasons of logistics or feasibility.

### The two-variable reduction

Given a predetermined number of sampled quanta within each measurement occasion, the general optimization problem above is reduced to the two-variable problem of identifying optimal values of *n*_*s *_and *n*_*d*_. This allows graphical illustrations of the problem and its solutions. It also opens for further simplification into one-variable optimisation problems, which in many cases can be solved explicitly, as shown in the results section.

The two-variable problem takes the form:(3)

with respect to *n*_*s*_*, n*_*d*_; subject to the constraint:(4)

In these equations, the terms  and  have been substituted into the three-variable expressions of mean exposure variance (equation (1)) and cost (equation (2)), respectively. This notation emphasizes that the specific variance of an exposure estimate obtained at one measurement occasion, *s*^*2*^_*μWD*_, and the cost of collecting data within each occasion, *c*_*q*_, are no longer allowed to vary.

In principle, the two-variable problem can be solved by applying constrained optimization techniques, i.e. by employing the problem's Lagrange function (e.g. [56]). As an alternative, the budget constraint, equation (4), can be substituted into the objective function, equation (3), so as to get a new objective function, which expresses the variance  as a function of only one variable, be it either *n*_*s *_or *n*_*d*_. This approach relies on the prerequisite that any solution to the optimization problem entails that the entire budget R is consumed. In that case, the budget constraint (equation (4)) can be replaced by an equality:(4a)

Isolating *n*_*s *_or *n*_*d *_from equation (4a), followed by substitution into equation (3), yields a one-variable objective function, , with *i *= *s *or *i *= *d*. This function can be examined using standard methodologies for identifying and illustrating possible local minima within a specified choice set. The resulting optimal value of either *n*_*s *_or *n*_*d *_can then be entered into the budget constraint to get the optimal value of the other variable.

### The one-variable substitution approach

The core challenge in the substitution approach outlined in the previous section is to identify that exposure assessment strategy in the choice set defined by the budget constraint for which the objective function, i.e. equation (3) with substituted *n*_*s *_or *n*_*d*_, has its minimal value. This can, in principle, be accomplished by determining the derivative of the objective function and finding its roots.

**Figure 2:**
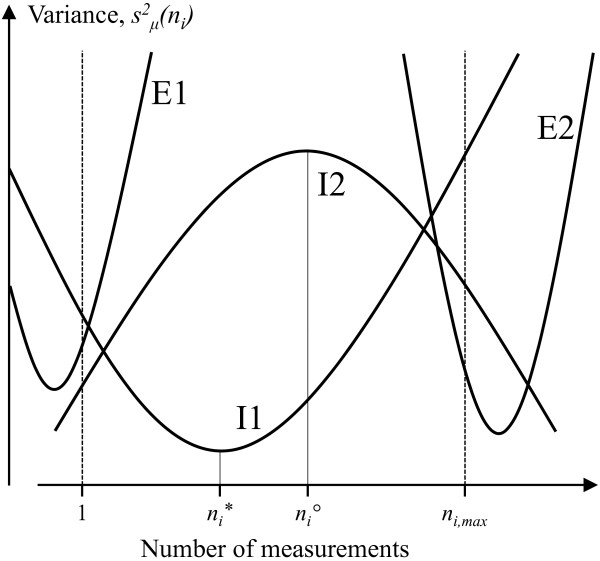
**Principally different cases of local extremes of the one-variable objective variance function**. The boundaries of possible resource investment, i.e. the choice set, are given by *n*_*i *_= 1 and *n*_*i *_= *n*_*i,max*_. In I1, the variance function has a local minimum at *n*_*i *_= *n*_i_*; this is an interior optimal solution with minimal variance. For I2, the variance function also has an interior zero derivative, at *n*_*i *_= *n*_*i*_*°*, but this solution maximizes the variance and is therefore not useful. In cases E1 and E2, the local extreme of the variance function lies below and above the choice set, respectively. In these cases, minimal variance is obtained at the lower (E1) and upper (E2) choice set boundary.

Figure 2 illustrates four principally different cases of how the objective variance function may look as a function of invested resources. At the lower boundary of the choice set, all resources are spent on one unit of *n*_*i*_, and at the upper boundary on as many *n*_*i *_as allowed by the budget, *n*_*i,max*_. Thus, if *i = s*, these two boundaries correspond to allocating as many measurement occasions as possible to one subject, and obtaining measurements at one occasion from as many subjects as possible.

As a general procedure, the optimal *n*_*i *_for a given budget can be found by comparing the performance obtained: (1) at the lower boundary of the choice set, i.e. using *n*_*i *_= 1, (2) at the upper boundary of the choice set, i.e. with *n*_*i *_= *n*_*i,max*_, and (3) entering values of *n*_*i*_, if any, in the interior of the choice set, 1 ≤ *n*_*i *_≤ *n*_*i*,max_, for which .

Thus, examining the properties of the objective function, , at the boundaries of the choice set is an appropriate first step for identifying the optimal allocation of resources. Provided that the objective function has one unique minimum, i.e. that the objective function is convex (I1, E1 and E2 in figure 2), a necessary, but also sufficient, condition for the optimum to be internal (case I1) is that  and . The exact location of the internal minimum can then be retrieved in a second step. The basic shape of the objective function can be determined by examining its second-order derivative. If this derivative is positive, the function is convex; if not it is concave (case I2), and the optimal strategy will be at one of the choice set boundaries.

If a convex objective function does not have an internal minimum, as in cases E1 and E2 in figure 2, the optimal strategy is represented by the boundary of the choice set. In case E1, which occurs if , the optimal strategy is to set *n*_*i *_= 1, that is, collect data from only one subject (if *i *= *s*), or having only one measurement occasion per subject (if *i *= *d*). Case E2 is characterized by a decreasing objective function at *n*_*i *_= *n*_max_, i.e.. In this case, if *i *= *s*, the best choice will be to measure as many subjects as possible and hence only one occasion per subject, or, if *i *= *d*, to collect data for as many occasions as possible from only one subject.

## Results

Below, procedures for determining optimal sampling strategies are developed using the one-variable substitution approach described above. Procedures will be stratified according to the sizes of *α *and *β*, which determine the shape of the cost function (equation (4a)), and hence the form of the substituted objective function,. For each combination of *α *and *β*, the objective function is examined, and the boundaries of the choice set determined. Procedures for determining whether the objective function is convex (cases I1, E1 and E2 in figure 2) or concave (case I2) are described where needed. For convex functions, explicit rules are, if possible, developed for when (case I1) and when not (cases E1, E2) the optimal measurement allocation occurs within the choice set. Finally, procedures for identifying an optimal sampling strategy inside the choice set (case I1) are described.

### Case A: α = 1, β = 1

In this case, the marginal costs of including another subject or measurement occasion are both independent of the number of previously included subjects and occasions. Thus, the cost function is linear at both of these stages.

#### Case A; substitution and objective function

With *α *= *β *= 1, the budget constraint (equation 4a) can be expressed as:(5)

Substituting this expression for *n*_*d *_in equation (3) gives the corresponding objective function:(6)

Taking the derivative with respect to *n*_*s *_yields:(7)

Setting , equation (7) can be expressed as:(7a)

This one-variable objective function is convex in *n*_*s*_, since the derivative of equation (7a) is positive for all *n*_*s *_in the choice set.

#### Case A; boundaries of the choice set

With *α *= *β *= 1, the choice set boundaries in terms of *n*_*s *_are *n*_*s *_= 1 and ; the latter obtained by setting *n*_*d *_= 1 in the budget constraint, equation (4a), and solving for *n*_*s*_.

At *n*_*s *_= 1, equation (7a) takes the form: . Thus, a positive derivative at *n*_*s *_= 1 occurs when:(8)

This gives a necessary and sufficient condition that the optimal allocation of measurements is obtained with *n*_*s *_= 1, and hence with  measurement occasions per subject.

At the other boundary, , the derivative of the objective function is:

This derivative is negative only when the first term in the numerator is negative, i.e., or rearranged:(9)

This is the necessary and sufficient condition for the optimal allocation being to choose the maximal affordable number of subjects, , and measure on one occasion for each of these. Notably, condition (9) is independent of the budget *R*. Also, unless  is zero, the condition is always valid if *π*_*s *_= 0, that is if the recruitment of subjects does not lead to any costs. Under case A, this implies that all measurement occasions entail the same cost, *π*_*d*_+*c*_*q*_, irrespective of how they are allocated between subjects. Thus, in this highly simplified case [38,39], the optimal strategy is always to measure on one occasion from each of as many subjects as allowed by the budget.

#### Case A; optimization inside the choice set

Setting the derivative of the variance function (7a) equal to zero yields:(10)

If this optimal value of *n*_*s *_is an interior solution, i.e., the corresponding number of measurement occasions per subject can be obtained by substitution of equation (10) into equation (4a):(11)

Thus, in this case the optimal number of measurement occasions per subject does not depend on the budget *R*.

The explicit solution derived above for the optimal set (*n*_*s*_*, n*_*d*_) can lead to non-integer values of one or both numbers. Since both are, by nature, discrete, a post-hoc procedure may be necessary in which integer sets of (*n*_*s*_*, n*_*d*_) close to the mathematically derived solution are entered into the budget constraint (equation (4)) to check that they are affordable, and into the objective function (equation (3)) to evaluate their statistical performance. For instance, if an interior *n*_*s *_determined by equation (10) is not an integer, the nearest larger and smaller integers are identified, and for each of those, at least two associated integer values of *n*_*d *_are determined that are larger and smaller than the value of *n*_*d *_derived by equation (11). The resulting affordable sets of (*n*_*s*_*, n*_*d*_) are then examined to identify the one resulting in the smallest mean exposure variance.

Table 1 summarizes the derived procedures for optimizing cost efficiency in case A, together with procedures for the other cases, as derived below.

**Table 1:** Summary of equations, in terms of their numbers in the running text, for identifying the optimal exposure assessment strategy

	Combination of *α *and *β*			
	
	A: *α *= 1;*β *= 1	**B: *α *= 1;*β***≠**1**	**C: *α***≠**1;*β *= 1**	**D: *α***≠**1;*β***≠**1**
Budget restriction	5	12	16	NA
Objective variance function; independent variable	6; *n*_*s*_	13; *n*_*d*_	17; *n*_*s*_	NA
Derivative of objective function	7 and 7a	14	18 and 18a	NA
Condition for choosing lower choice set boundary	8	15	19	NA
Condition for choosing upper choice set boundary	9	NA	NA	NA
Internal *n*_*s*_	10	NA	NA	NA
Internal *n*_*d*_	11	NA	NA	NA

Equations express the budget restriction, the objective function, its derivative, the conditions for the lower and upper boundaries of the choice set to be optimal solutions to the allocation of samples, and the interior solution to the optimization, if applicable. NA: no analytic solution available; numerical methods must be applied (see text).

### Case B: α = 1, β≠1

Case B entails constant marginal costs in the recruitment of new subjects but either increasing or decreasing marginal costs for organizing measurement occasions.

#### Case B; substitution and objective function

In case B, the one-variable problem is most easily solved if the objective function is rearranged so that *n*_*s *_is expressed as a function of *n*_*d*_. From the budget constraint, equation (4a), *n*_*s *_is isolated as:(12)

The corresponding objective function is:(13)

And its derivative:(14)

The objective function (equation (13)) is always convex for *β *≥ 2. For 1 <*β *< 2 it is convex if , and for *β *< 1, convexity requires  (proof, see appendix).

If none of these inequalities are fulfilled, the optimal measurement strategy will correspond to one of the choice set boundaries.

#### Case B; boundaries of the choice set

**Figure 3:**
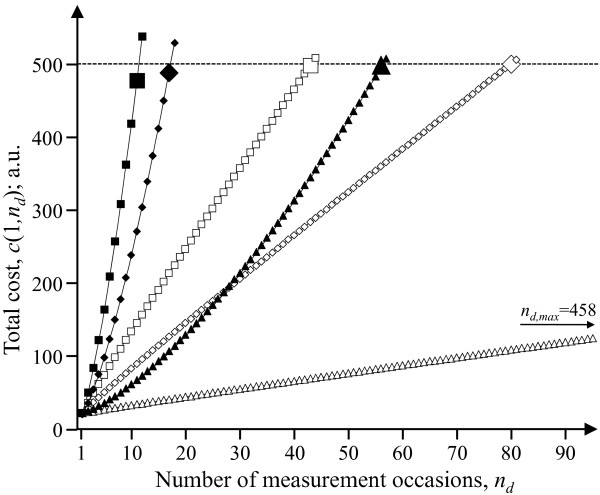
**Numerical determination of the upper boundary of the choice set in case B (α = 1, β≠1)**. For six different combinations of unit costs and size of the exponent β, the maximal possible number of measurement occasions, i.e. *n*_*d,max*_, for a single subject is identified under a budget constraint of 500 (arbitrary units). Squares, rhomboids, and triangles: (*π*_*s*_*, π*_*d*_*, c*_*q*_) = (2, 10, 10), (11, 5.5, 5.5), and (20, 1, 1), respectively. Open and closed symbols: *β *= 0.50 and *β *= 1.50, respectively. The value of *n*_*d,max *_in each scenario is indicated by an enlarged symbol.

The choice set boundaries in terms of *n*_*d *_are *n*_*d *_= 1 and *n*_*d *_= *n*_*d*,max_. The latter is found by setting *n*_*s *_= 1 in the budget constraint, equation (4a), and rearrange to get: . This equation does not have a closed-form solution for *n*_*d*_. In this case, *n*_*d*,max _can be determined numerically by calculating the cost, *c*(1, *n*_*d*_), when entering increasing values of *n*_*d *_in the cost function, equation (4), at *n*_*s *_= 1, that is:

*n*_*d*,max _is then the largest value of *n*_*d *_for which *c*(1, *n*_*d*_) ≤ *R*. Figure 3 illustrates an example of this procedure, for three different combinations of (*π*_*s*_, *π*_*d*_, *c*_*q*_) and two different levels of *β*, which will reappear in the collection of numerical examples.

At *n*_*d *_= 1, the derivative of the objective function, i.e. equation (14), is equal to:(14a)

which is positive under the following condition:(15)

Thus, for parameter sets obeying this inequality, the optimal sample allocation is to measure for one occasion on each of  subjects.

At the other boundary, *n*_*d *_= *n*_*d*,max_, the sign of the derivative of the objective function must be obtained by entering the numerically determined value of *n*_*d*,max _in equation (14). A negative  is then a necessary and sufficient condition for the optimal measurement strategy to be to choose one subject and measure record from that subject on *n*_*d*,max _occasions.

#### Case B; optimization inside the choice set

The objective function, equation (13), cannot be minimized using analytical methods, since  (cf. equation (14)) does not have a closed-form solution. Thus, a possible interior optimum must be located by entering all values of *n*_*d *_in the interval [1, *n*_*d*,max_] into the objective function and locate the minimal result. The corresponding optimal value of *n*_*s *_can be found by entering the identified optimal value of *n*_*d *_in equation (12).

### Case C: α≠1, β = 1

In case C, all measurement occasions for a particular subject can be organized at the same cost, while the cost of recruiting additional subjects changes with their numbers.

#### Case C; substitution and objective function

In case C, the one-variable problem is most easily solved if the objective function is rearranged to express *n*_*d *_as a function of *n*_*s*_. Isolating *n*_*d *_in the budget constraint, equation (4a), gives(16)

And hence the objective variance function in terms of *n*_*s *_is:(17)

Taking the derivate with respect to *n*_*s *_yields:(18)

Setting , this can be expressed as:(18a)

It is straightforward to verify that this function is convex in *n*_*s *_and, hence, has one unique minimum.

#### Case C; boundaries of the choice set

The choice set boundaries in this case are *n*_*s *_= 1 and *n*_*s *_= *n*_*s*,max_. The latter is found by setting *n*_*d *_= 1 in the budget constraint, equation (4a), and solving for *n*_*s*_. This leads to the equation:  which does not have a closed-form solution. Thus, similar to the determination of *n*_*d*,max _in case B above, *n*_*s*,max _must be determined by entering increasing values of *n*_*s *_in the cost function  until reaching the largest value of *n*_*s *_for which *c*(*n*_*s*_, 1) ≤ *R*.

At the boundary *n*_*s *_= 1, the derivative of the objective function, c.f. equation 18a, is:  A necessary and sufficient condition for choosing *n*_*s *_= 1, and hence  (cf. equation (16); if necessary truncated to the nearest smaller integer), is derived by rearranging the inequality  to give:(19)

At the other boundary, *n*_*s*,max_, the sign of the derivative of the objective function must be determined numerically by entering the *n*_*s*,max _identified above into equation (18a). If the sign is negative, *n*_*s*,max _is the optimal number of subjects, and each should be recorded for one occasion.

#### Case C; optimization inside the choice set

In case C, the equation  (cf. equation (18a)) has no closed-form solution. Thus, an interior solution to the optimization must be identified by entering all *n*_*s *_in the interval [1, *n*_*s*,max_] into the objective function, i.e. equation (17), and locate the minimal variance. After having identified the optimal *n*_*s*_, the corresponding *n*_*d *_can be found by solving equation (16).

### Case D: α≠1, β≠1

In case D, neither *n*_*s *_nor *n*_*d *_can be expressed as a function of the other on basis of the budget constraint. Thus, a one-variable problem cannot be formulated in explicit terms, and, consequently, no analytical expressions can be developed, neither for the derivative of the objective function, nor for boundary conditions, nor for possible interior solutions. Therefore, the optimal choice of the number of subjects and measurement occasions has to be identified by means of a numerical procedure, such as the following:

(1) For *n*_*s *_= 1, the cost function, equation (4), is .

In this function, increasing *n*_*d *_-values are entered, up to largest possible value, *n*_*d*,max_, for which *c*(1, *n*_*d*_) ≤ *R*;

(2) The values (*n*_*s*_, *n*_*d*_) = (1, *n*_*d*,max1_) are entered into the objective function, equation (3), i.e. , and the resulting value is noted.

(3) These two steps are repeated for *n*_*s *_= 2, corresponding to the cost function, thus obtaining the value of 

(4) Subsequent values of  are derived using this same procedure for stepwise increasing *n*_*s*_, until reaching the largest possible *n*_*s *_allowed by the budget.

(5) By inspecting the set of values of , which all entail costs as close as possible to the budget constraint *R*, the combination of *n*_*s *_and *n*_*d *_offering the smallest variance can be identified.

**Figure 4:**
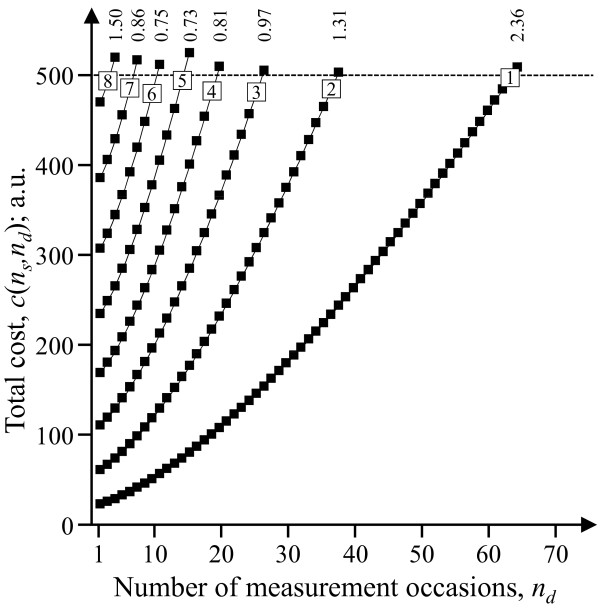
**Numerical procedure for determining the optimal exposure assessment strategy in case D (α≠1, β≠1)**. For increasing values of *n*_*s *_as indicated inside the open symbols in each curve, the maximal number of measurement occasions, i.e. *n*_*d*,max*ns*_, allowed by a budget of 500 (arbitrary units) is identified, as marked by open symbols. The resulting statistical performance, i.e. *s*^*2*^_*μ*_(*n*_*s*_*, n*_*d*,max*ns*_), is shown above each curve. In the illustrated case, (*n*_*s*_*, n*_*d*_) = (5, 12) was the optimal allocation. The illustration refers to a scenario with (*s*^*2*^_*BS*_*, s*^*2*^_*BD*_*, s*^*2*^_*μWD*_) = (2, 10, 10), (*π*_*s*_*, π*_*d*_*, c*_*q*_) = (20, 1, 1), and (α, β) = (1.50, 1.50).

Figure 4 illustrates the numerical procedure for identifying the maximal possible value of *n*_*d *_at increasing values of *n*_*s*_, and the resulting variance of the exposure mean. Since the values of *n*_*s *_and *n*_*d *_are discrete, and hence even the corresponding total cost *c*(*n*_*s*_*, n*_*d*_), it may happen that the optimal measurement strategy does not consume the entire budget *R*. For instance, the optimal strategy (*n*_*s*_*, n*_*d*_) = (5, 12) identified in figure 4 only utilizes 98.3% of the allowed resources.

### Numerical examples

Using the procedures developed above, optimal sampling strategies were identified for 225 scenarios representing different combinations of costs and variance components, and different marginal costs of recruiting new subjects and organizing more measurement occasions, as expressed through *α *and *β *(table 2). Unit costs *π*_*s*_*, π*_*d *_and *c*_*q *_were selected to illustrate large, medium and small costs of recruiting subjects relative to obtaining measurements on each of them, and the sets of variance components ,  and  represent large, medium and small between-subjects to within-subject variance ratios. Parameter values were chosen so that the total cost of assessing the exposure of one subject at one occasion (cf. equation (4)) as well as the resulting mean exposure variance (cf. equation (3)) takes the same numerical value (22) in all scenarios. In all scenarios, the budget R was constrained at 500 (arbitrary units). In median, the 225 strategies utilized 97.9% of the allowed budget (5^th^-95^th ^percentile range: 92.3% to 100.0%).

**Table 2:** Optimal sampling strategies (*n*_*s*_*, n*_*d*_) and the resulting mean exposure variance *s*^*2*^_*μ *_(cf. equation (3)) at different combinations of variance components (*s*^*2*^_*BS*_*,s*^*2*^_*BD*_*,s*^*2*^_*μWD*_; sections **a**-**c**), unit costs (*π*_*s*_*, π*_*d*_*, c*_*q*_), and exponents *α *and *β *describing the shape of the relationship between costs and number of measurements (cf. equation (4))

**a. (*s***^***2***^_***BS***_***, s***^***2***^_***BD***_***, s***^***2***^_***μWD***_**) = (2, 10, 10)**											
	***α***:	0.50		0.75		1.00		1.25		1.50	
		**(*n***_***s***_***, n***_***d***_**)**	***s***^***2***^_***μ***_	**(*n***_***s***_***, n***_***d***_**)**	***s***^***2***^_***μ***_	**(*n***_***s***_***, n***_***d***_**)**	***s***^***2***^_***μ***_	**(*n***_***s***_***, n***_***d***_**)**	***s***^***2***^_***μ***_	**(*n***_***s***_***, n***_***d***_**)**	***s***^***2***^_***μ***_
(*π*_*s*_*, π*_*d*_*, c*_*q*_)	*β*										
(2, 10, 10)	0.50	(14, 2)	0.86	(14, 2)	0.86	(10, 3)	0.87	(13, 2)	0.92	(9, 3)	0.96
	0.75	(24, 1)	0.92	(13, 2)	0.92	(9, 3)	0.96	(12, 2)	1.00	(8, 3)	1.08
	1.00	(24, 1)	0.92	(23, 1)	0.96	(22, 1)	1.00	(11, 2)	1.09	(10, 2)	1.20
	1.25	(24, 1)	0.92	(23, 1)	0.96	(22, 1)	1.00	(20, 1)	1.10	(17, 1)	1.29
	1.50	(24, 1)	0.92	(23, 1)	0.96	(22, 1)	1.00	(20, 1)	1.10	(17, 1)	1.29
(11, 5.5, 5.5)	0.50	(17, 3)	0.51	(15, 3)	0.58	(11, 4)	0.64	(7, 7)	0.69	(5, 10)	0.80
	0.75	(22, 2)	0.55	(14, 3)	0.62	(10, 4)	0.70	(6, 7)	0.81	(6, 6)	0.89
	1.00	(39, 1)	0.56	(18, 2)	0.67	(9, 4)	0.78	(8, 4)	0.88	(6, 5)	1.00
	1.25	(39, 1)	0.56	(32, 1)	0.69	(14, 2)	0.86	(7, 4)	1.00	(6, 4)	1.17
	1.50	(39, 1)	0.56	(32, 1)	0.69	(13, 2)	0.92	(6, 4)	1.17	(5, 4)	1.40
(20, 1, 1)	0.50	(49, 5)	0.12	(27, 7)	0.18	(14, 12)	0.26	(8, 23)	0.36	(6, 28)	0.45
	0.75	(52, 4)	0.13	(21, 9)	0.20	(13, 12)	0.28	(8, 19)	0.38	(6, 23)	0.48
	1.00	(80, 2)	0.15	(26, 5)	0.23	(13, 9)	0.32	(8, 14)	0.43	(6, 17)	0.53
	1.25	(74, 2)	0.16	(27, 4)	0.26	(13, 7)	0.37	(8, 10)	0.50	(6, 12)	0.61
	1.50	(134, 1)	0.16	(29, 3)	0.30	(12, 6)	0.44	(9, 6)	0.59	**(5, 12)**	**0.73**

**b. (*s*^*2*^_*BS*_*, s*^*2*^_*BD*_*, s*^*2*^_*μWD*_) = (11, 5.5, 5.5)**											
	***α***:	**0.50**		**0.75**		**1.00**		**1.25**		**1.50**	
		**(*n***_***s***_***, n***_***d***_**)**	***s***^***2***^_***μ***_	**(*n***_***s***_***, n***_***d***_**)**	***s***^***2***^_***μ***_	**(*n***_***s***_***, n***_***d***_**)**	***s***^***2***^_***μ***_	**(*n***_***s***_***, n***_***d***_**)**	***s***^***2***^_***μ***_	**(*n***_***s***_***, n***_***d***_**)**	***s***^***2***^_***μ***_

(2, 10, 10)	0.50	(24, 1)	0.92	(23, 1)	0.96	(22, 1)	1.00	(20, 1)	1.10	(17, 1)	1.29
	0.75	(24, 1)	0.92	(23, 1)	0.96	(22, 1)	1.00	(20, 1)	1.10	(17, 1)	1.29
	1.00	(24, 1)	0.92	(23, 1)	0.96	(22, 1)	1.00	(20, 1)	1.10	(17, 1)	1.29
	1.25	(24, 1)	0.92	(23, 1)	0.96	(22, 1)	1.00	(20, 1)	1.10	(17, 1)	1.29
	1.50	(24, 1)	0.92	(23, 1)	0.96	(22, 1)	1.00	(20, 1)	1.10	(17, 1)	1.29
(11, 5.5, 5.5)	0.50	(39, 1)	0.56	(32, 1)	0.69	(22, 1)	1.00	(12, 2)	1.38	(9, 2)	1.83
	0.75	(39, 1)	0.56	(32, 1)	0.69	(22, 1)	1.00	(12, 2)	1.38	(9, 2)	1.83
	1.00	(39, 1)	0.56	(32, 1)	0.69	(22, 1)	1.00	(15, 1)	1.47	(9, 2)	1.83
	1.25	(39, 1)	0.56	(32, 1)	0.69	(22, 1)	1.00	(15, 1)	1.47	(8, 2)	2.06
	1.50	(39, 1)	0.56	(32, 1)	0.69	(22, 1)	1.00	(15, 1)	1.47	(8, 2)	2.06
(20, 1, 1)	0.50	(134, 1)	0.16	(44, 2)	0.38	(19, 4)	0.72	(11, 6)	1.17	(7, 14)	1.68
	0.75	(134, 1)	0.16	(43, 2)	0.38	(18, 4)	0.76	(11, 5)	1.20	(7, 12)	1.70
	1.00	(134, 1)	0.16	(42, 2)	0.39	(19, 3)	0.77	(11, 4)	1.25	(7, 9)	1.75
	1.25	(134, 1)	0.16	(40, 2)	0.41	(18, 3)	0.81	(10, 5)	1.32	(7, 7)	1.80
	1.50	(134, 1)	0.16	(53, 1)	0.42	(20, 2)	0.83	(11, 3)	1.33	(7, 5)	1.89

**c. (*s*^*2*^_*BS*_*, s*^*2*^_*BD*_*, s*^*2*^_*μWD*_) = (20, 1, 1)**											
	***α***:	**0.50**		**0.75**		**1.00**		**1.25**		**1.50**	
		**(*n***_***s***_***, n***_***d***_**)**	***s***^***2***^_***μ***_	**(*n***_***s***_***, n***_***d***_**)**	***s***^***2***^_***μ***_	**(*n***_***s***_***, n***_***d***_**)**	***s***^***2***^_***μ***_	**(*n***_***s***_***, n***_***d***_**)**	***s***^***2***^_***μ***_	**(*n***_***s***_***, n***_***d***_**)**	***s***^***2***^_***μ***_

(2, 10, 10)	0.50	(24, 1)	0.92	(23, 1)	0.96	(22, 1)	1.00	(20, 1)	1.10	(17, 1)	1.29
	0.75	(24, 1)	0.92	(23, 1)	0.96	(22, 1)	1.00	(20, 1)	1.10	(17, 1)	1.29
	1.00	(24, 1)	0.92	(23, 1)	0.96	(22, 1)	1.00	(20, 1)	1.10	(17, 1)	1.29
	1.25	(24, 1)	0.92	(23, 1)	0.96	(22, 1)	1.00	(20, 1)	1.10	(17, 1)	1.29
	1.50	(24, 1)	0.92	(23, 1)	0.96	(22, 1)	1.00	(20, 1)	1.10	(17, 1)	1.29
(11, 5.5, 5.5)	0.50	(39, 1)	0.56	(32, 1)	0.69	(22, 1)	1.00	(15, 1)	1.47	(10, 1)	2.20
	0.75	(39, 1)	0.56	(32, 1)	0.69	(22, 1)	1.00	(15, 1)	1.47	(10, 1)	2.20
	1.00	(39, 1)	0.56	(32, 1)	0.69	(22, 1)	1.00	(15, 1)	1.47	(10, 1)	2.20
	1.25	(39, 1)	0.56	(32, 1)	0.69	(22, 1)	1.00	(15, 1)	1.47	(10, 1)	2.20
	1.50	(39, 1)	0.56	(32, 1)	0.69	(22, 1)	1.00	(15, 1)	1.47	(10, 1)	2.20
(20, 1, 1)	0.50	(134, 1)	0.16	(53, 1)	0.42	(22, 1)	1.00	(12, 2)	1.75	(8, 3)	2.58
	0.75	(134, 1)	0.16	(53, 1)	0.42	(22, 1)	1.00	(12, 2)	1.75	(8, 3)	2.58
	1.00	(134, 1)	0.16	(53, 1)	0.42	(22, 1)	1.00	(12, 2)	1.75	(8, 2)	2.63
	1.25	(134, 1)	0.16	(53, 1)	0.42	(22, 1)	1.00	(12, 2)	1.75	(8, 2)	2.63
	1.50	(134, 1)	0.16	(53, 1)	0.42	(22, 1)	1.00	(12, 1)	1.83	(8, 2)	2.63

In all cases, the available budget *R *was set at 500. The determination of the boldface strategy in section **a **was illustrated in figure 4.

As illustrated in table 2, the optimally cost-efficient strategy in many scenarios is to obtain data on one occasion from as many subjects as possible. In particular, this applies when *s*^*2*^_*BS *_is "large" relative to *s*^*2*^_*BD *_and *s*^*2*^_*μWD *_(table 2c), and even when *s*^*2*^_*BS *_is similar to (*s*^*2*^_*BD*_+*s*^*2*^_*μWD*_) if *π*_*s *_is also equal to or smaller than (*π*_*d*_+*c*_*q*_) (table 2b). In these cases, the principle of measuring from as many subjects as possible is valid irrespective of whether cost functions are linear or not, i.e. irrespective of the sizes of *α *and *β*.

Considerable deviations from the principle of collecting data from as many subjects as possible do, however, occur; the most extreme examples appearing when *s*^*2*^_*BS *_is "small" relative to *s*^*2*^_*BD *_and *s*^*2*^_*μWD *_*and π*_*s *_is "large" compared to (*π*_*d*_+*c*_*q*_) *and α *is "large" (bottom right corner of table 2a). The combination of a "small" variance between subjects and "large" costs associated with recruiting subjects also leads to the optimal sampling strategy being particularly sensitive to non-linearities in costs. Thus, with (*s*^*2*^_*BS*_*, s*^*2*^_*BD*_*, s*^*2*^_*μWD*_) = (2, 10, 10) and (*π*_*s*_*, π*_*d*_*, c*_*q*_) = (20, 1, 1), a linear cost function implies an optimal sampling strategy of (*n*_*s*_*, n*_*d*_) = (13, 9) (table 2a), while the deviations of *α *and *β *from 1 illustrated in table 2 result in optimal strategies (*n*_*s*_*, n*_*d*_) ranging from (5, 12) to (49, 5), and corresponding variances *s*^*2*^_*μ *_between 0.12 and 0.73. In contrast, with (*s*^*2*^_*BS*_*, s*^*2*^_*BD*_*, s*^*2*^_*μWD*_) = (20, 1, 1) and (*π*_*s*_*, π*_*d*_*, c*_*q*_) = (2, 10, 10) (table 2c), the most extreme non-linear cost functions lead to sampling strategies, (*n*_*s*_*, n*_*d*_) = (24, 1) and (*n*_*s*_*, n*_*d*_) = (17, 1), which do not deviate much from the optimal strategy in the linear case, (*n*_*s*_*, n*_*d*_) = (22, 1), and only result in moderate differences in variance.

While not illustrated in table 2, a larger total budget leads to a wider occurrence of the optimal strategy being to collect data on one occasion per subject. Thus, with a budget of 500, 135 of the 225 scenarios illustrated in table 2 imply that data should be collected according to this principle; if the budget is increased to 1000, this count increases to 139. However, in 3 cases the optimal strategy changes in the opposite direction, i.e. into collecting data on more than one occasion per subject. This was caused by irregularities due to the effect of *n*_*s *_and *n*_*d *_needing to be integers. With a decreasing budget, one-occasion-per-subject optima get rarer, as expected, but irregularities occur more often.

Even if non-linearities in cost functions may not affect the *principle *of how to allocate measurements at many combinations of unit costs and variance components, the size of *α *is always important to the eventual *size *of the data set, and therefore to the precision of the eventual mean exposure estimate. In contrast, the size of *β *is only important if the optimal strategy implies, or is close to implying, measurements from more than one occasion per subject, that is when *s*^*2*^_*BS *_is "small" relative to *s*^*2*^_*BD *_and *s*^*2*^_*μWD *_(table 2a), but even when *s*^*2*^_*BS *_is similar to (*s*^*2*^_*BD*_+*s*^*2*^_*μWD*_) if *π*_*s *_is also larger than (*π*_*d*_+*c*_*q*_) (table 2b). This is an expected result, since the cost of setting up measurement occasions is independent of *β *at *n*_*d *_ = 1 (cf. equation (4)). Thus, when analyzing whether an intended exposure assessment strategy, constrained by budgets, will lead to a sufficient statistical performance, access to a valid estimate of *α *is generally more important than knowing the exact size of *β*.

While the size of *β *is not always important to size of the optimal data set, the best statistical performance at any specific combination of (*s*^*2*^_*BS*_*, s*^*2*^_*BD*_*, s*^*2*^_*μWD*_) and (*π*_*s*_*, π*_*d*_*, c*_*q*_) will always be obtained with small sizes of *α *and *β*; in table 2 exemplified by (*α, β*) = (0.50, 0.50). This is a reasonable result, since small *α *and *β *entail small marginal costs of including more subjects and more measurement occasions.

Although not illustrated in table 2, the effects on statistical performance of deviating from the optimal choice of (*n*_*s*_*, n*_*d*_), but still using the entire budget, were also investigated. In certain cases, deviations did not lead to any particular reduction of performance. For instance, with (*s*^*2*^_*BS*_*, s*^*2*^_*BD*_*, s*^*2*^_*μWD*_) = (2, 10, 10), (*π*_*s*_*, π*_*d*_*, c*_*q*_) = (20, 1, 1), and (*α, β*) = (0.75, 0.75), the optimal strategy is to choose (*n*_*s*_*, n*_*d*_) = (21, 9), resulting in a variance of 0.20 (cf. table 2a). However, all strategies with *n*_*s *_in the range between 15 and 32, and corresponding values of *n*_*d*,max*ns *_ranging from 15 to 4 as allowed by the budget, resulted in variances of 0.22 or less, except for the strategy (30, 4) which gave a variance of 0.23 because it only managed to utilize 92% of the available budget. In other cases, performance was more sensitive to non-optimal choices of (*n*_*s*_*, n*_*d*_). Again using (*s*^*2*^_*BS*_*, s*^*2*^_*BD*_*, s*^*2*^_*μWD*_) = (2, 10, 10) and (*α, β*) = (0.75, 0.75), the optimal strategy with (*π*_*s*_*, π*_*d*_*, c*_*q*_) = (2, 10, 10) is now (*n*_*s*_*, n*_*d*_) = (13, 2), resulting in a variance of 0.92 (table 2a). In this case, all strategies allowed by the budget besides the nearest neighbour, (*n*_*s*_*, n*_*d*_) = (12, 2), gave variances of 1.09 or more, i.e. at least 18% larger than the optimum.

## Discussion

As illustrated by the numerical examples in table 2, a large ratio of between-subjects to within-subject variance generally implies that the optimal allocation *principle *is to collect data on one occasion from as many subjects as allowed by the budget. This also applies when between-subjects and within-subject variances are of similar size, unless the unit cost of recruiting subjects is large relative to that of setting up measurement occasions. In these cases, non-linearity in the cost functions does not influence the optimal allocation principle; only the eventual *size *of the data set allowed by budgets. However, at a large relative recruitment cost combined with a small between-subjects to within-subject variance ratio, and in particular if the total budget is also small, the optimal sampling strategy may consist in approaching only a few subjects on several occasions each, and the strategy is very sensitive to non-linearities in cost functions. Non-linearities in subject recruitment costs always have a clear influence on the *size *of the optimal data set, while non-linearities in costs for setting up measurement occasions are important only in cases when the optimal strategy includes multiple measurements per subject.

### Representativeness

#### Statistical model

The present study investigated a hierarchical, nested measurement model with three stages as used in a majority of previous studies of the effects of random measurement error on statistical properties and efficiency in exposure assessment (e.g. [2,12,26-28]). Even though the application exemplified in the paper refers to subjects, measurement occasions within subjects, and measurement units within occasions, the generic results are applicable also to other sources of exposure variability that can be described by a hierarchical model. This includes the case of data processing and analysis adding "post-sampling" costs and also some methodological variance to each collected exposure sample, thus modifying the sizes of *c*_*q *_(equation (4)) and  (equation (3)), respectively. Also, the present study addressed, as most other studies, the case of balanced data sampling, i.e. that the same number of measurement units are collected during each of the same number of occasions from each subject [23]. While the assumption of a balanced, hierarchical model facilitates mathematical derivation of optimal measurement strategies, cost-efficiency needs to be investigated even for more complicated models, for instance designs including crossed components [11,29]. In particular, the effects of unbalancedness, which is probably a very frequent incident in epidemiologic research, need to be addressed in further studies. Unbalancedness has been shown both mathematically [23,57] and empirically [58] to reduce statistical efficiency, and will thus also influence cost-efficiency.

During the last decade, powerful statistical techniques have been developed to analyse exposure variability and its determinants using so-called mixed-effect modelling [30-33,59]. While mixed model analyses have predominantly been used to identify exposure targets for effective prevention and intervention, they also represent a challenging opportunity to develop exposure assessment strategies that are both "cheap" and statistically efficient. As an example, several occupational studies have proposed or implemented the idea of estimating full-shift job exposures by combining observed or self-reported time proportions of tasks in the job with task exposures from a data base [60-65]. In some studies, the task-based estimates appeared easy to obtain and, at the same time, well correlated with "true" job exposures (e.g. [66]), while other studies indicate that task-based procedures can also be grossly inefficient [64,65]. Some attention has been given to developing mathematical principles for assessing the statistical performance of task-based exposure modelling [34,67], but no studies have so-far, to our knowledge, addressed if task-based assessment can, indeed, be cost-efficient as compared to direct measurement of job exposures, and if so, on which conditions. A similar concern can be raised with respect to other techniques for combining exposure information from different sources into a "hybrid" estimate of some exposure metric [68]. The approach can be statistically informative [68], but might also entail costs to the extent that the trade-off between efficiency and resource consumption is disadvantageous as compared to measuring "true" exposures directly.

#### Statistical performance criterion

The present study addressed the objective of obtaining a precise estimate of the exposure mean value in a group of subjects (cf. equation (3)), the reason being that precision of the mean is a decisive factor for the usefulness of exposure surveys, and for statistical power in studies comparing conditions and groups. Other measures of statistical performance will, however, be of interest in other types of epidemiologic research, and thus need attention in future cost-efficiency research. A particularly important example is the size of bias and/or precision in a regression of outcome on exposure [19-22]. Since both bias and precision can, under a number of assumptions, be expressed as mathematical functions of variance components and the number of measurements [18], it might be possible to develop closed-form solutions to the problem of finding optimally cost-efficient measurement strategies, but this has not so-far been pursued. Another example that an exposure assessment strategy may have another purpose than producing a satisfying group exposure mean is standard surveillance of compliance with occupational exposure limits (OEL). First, the assessment focuses on individuals rather than groups, and second, the strategy needs assure that both the individual mean and the probability that single exposure values exceed the OEL is determined with a satisfying certainty [16,17]. Still another relevant measure of statistical performance for several purposes is the size of the standard reliability coefficient (ICC), i.e. the relationship between exposure variability in data sets with and without (random) measurement error [41].

Obviously, both for regression metrics, exceedance, and ICCs, optimally cost-efficient exposure assessment strategies may deviate from those driven by the objective of obtaining precise exposure means, as illustrated by two studies on optimal measurement allocation in reliability studies [69,70].

A particularly challenging situation comes up if the exposure assessment strategy has two simultaneous, yet conflicting objectives. For instance, the researcher may, at the same time, wish to get a precise estimate of a group mean exposure, but also a good estimate of exposure variance components between and within workers. This is a likely scenario if the specific exposure variability of the addressed occupational group is *a priori *insufficiently known, and the exposure data collection is viewed as an opportunity to get updated data on this variability, together with a documentation of the group mean exposure. Determination of variance components requires, as a minimum, duplicate samples at each stage of the measurement model [5], and this may often *not *be an optimally cost-efficient strategy if the objective is to get a precise group mean (cf. table 2a-c; cases with *n*_*d *_= 1). Thus, the researcher faces the decision of whether a certain loss in information on the group mean is an acceptable "price" of getting some information on exposure variability. While the numerical trade-off between these two types of information, conditional on a restricted budget, may be resolved in future research, the final decision of which sampling allocation to prefer is an issue beyond mathematical procedures.

#### Recruitment capabilities and cost functions

While presenting a novel approach in allowing recruitment capabilities and, as a consequence, the corresponding cost functions to be non-linear, the present study only addressed the case when non-linearities can be expressed using homogeneous functions. This type of non-linear production capabilities is often assumed in economics research, but other types of mathematical relationships may, obviously, be appropriate. Even cost functions that do not follow monotonous mathematical rules may apply, as illustrated by the example in Duan and Mage [42], where the basic shape of the cost function changes with the number of measurements, and by some examples in Cochran's excellent textbook [47]. We claim a strong need to bring forward more empirical evidence to suggest the appropriate *shape *of cost functions in exposure assessment; and if power relationships are, indeed, supported, to indicate reasonable sizes of the exponents *α *and *β*. Hypothetically, the recruitment of subjects could entail increasing marginal costs (*α*>1), as if additional time has to be devoted to persuading initially reluctant participants, but also decreasing costs (*α*<1), as if the first subjects are hard to recruit but their skeptic colleagues, taking after them, will then readily participate. Also, both increasing and decreasing marginal costs for organizing measurement occasions can be envisaged, as if a measurement equipment wears down over time and needs to be in place longer to provide a certain amount of data (*β *>1), or if a subject gets more and more accustomed to measurement preparations and thus less time consuming (*β *<1). As a tentative conjecture, however, considerable deviations of *α *from 1 are more likely to occur than deviations of *β*. In addition to the need for empirical data describing the *shape *of cost functions, information is also required concerning the *size *of unit costs for measuring at different stages; very little data has been reported in occupational or environmental epidemiology [37,43]. This stands in a striking contrast to the abundance of data on variance components for a multitude of occupational and environmental exposures, showing that the size of and relationship between exposure variabilities at different stages of measurement, e.g. subjects and occasions within subjects, differ widely between settings and exposure agents [3,9-11,25,71,72].

In the present study, optimization procedures were developed using a total cost model including only variable cost components (equation (4)). Other studies have addressed even fixed costs, i.e. costs that do not depend on the number of measurements [41,43]. While fixed costs are, under a constrained budget, decisive to the resources left for allocating measurements, they cancel out in the course of the mathematical differentiation associated with the optimization procedure, and thus will not affect the eventual optimal allocation strategy [43]. It is, however, important to notice that the optimization procedures in the present paper all refer to budgets where possible fixed costs have already been accounted for.

#### Analytical vs. numerical optimization

A complete closed-form mathematical solution to cost-efficiency optimization was possible only when cost functions were linear, i.e. (*α, β*) = (1, 1), and in this case the allocation algorithms were consistent with previous studies [43,44,46,47]. When either *α *or *β *deviated from 1, neither the choice set boundaries nor an internal optimum could be explicitly determined, and if both deviated together, all optimization steps had to be performed using numerical methods. This suggests that explicit, formal expressions defining cost-efficient measurement allocations may only be obtainable if both cost functions and expressions of statistical performance are mathematically very simple. Thus, numerical optimization procedures might be the only alternative if, for instance, the objective (*in casu *variance) function contains not only nested components [11,29], or if the cost model does not express a straight-forward relationship with the number of measurements [42]. This points to the idea of basing all optimization on numerical methods and ignore explicit solutions even in those cases where they do exist. However, we believe that mathematical expressions as developed in this paper may still be helpful as a screening tool for deciding whether the optimal strategy needs further (numerical) consideration, or whether it is merely situated at the boundary of the choice set, as in those frequent cases where as many subjects as possible should be measured on one occasion each (cf. table 2).

### Sensitivity

#### The basic cost model

One important result of the present investigation was that for many combinations of unit costs and variance components, non-linear cost functions did not change the general principle stated by a linear model: to measure from as many subjects as possible on one occasion each (cf. table 2). Thus, under these particular circumstances, the principle of how to optimize exposure assessment was not sensitive to the cost model, even if the eventual size of the data set allowed by budget constraints was influenced by non-linearities in subject recruitment costs. At other combinations of variance components and unit costs, in particular when between-subject variability was small compared to within-subject variability and subject recruitment costs at the same time were large compared to costs for setting up measurement occasions, non-linearities did, however, strongly affect both the optimal allocation principle and the eventual statistical performance. While, as mentioned above, examples of small between- to within-subject ratios of variance are abundant in the literature, relative sizes of unit costs are largely unknown, and thus we do not consider it justified so-far to form an opinion on the actual occurrence of such sensitive scenarios.

#### Uncertainties in input parameters

The procedures developed in the present study for identifying optimal exposure assessment strategies, whether analytical or numerical, rely on known values of unit costs, exponents in the cost function, and variance components. However, in a specific epidemiologic study, all of these inputs need be based on estimates associated with some degree of uncertainty. Thus, the derived "optimal" exposure assessment strategy will, in itself, be uncertain. Similar to the issue of cost function sensitivity discussed above, the *principle *of how to optimize exposure assessment seem, however, to be very robust to changes in unit costs and variance components when between-subject variability is large compared to within-subject variability and subject recruitment costs are small or similar to costs for setting up measurement occasions (table 2). Even the *size *of the eventual data set is robust to changes in exposure variability, as long as recruitment costs are small (table 2). If, however, recruitment unit costs are large, both the allocation and size of the optimal strategy is highly sensitive to the size of variance components, especially if recruitment costs accelerate with the number of subjects (*α*>1).

Even when closed-form solutions are available for estimating the optimal choice of subjects and measurement occasions (equations (10) and (11)), a corresponding analytical expression of the uncertainty of these estimates may not be readily available. Optimization using numerical procedures evidently precludes any explicit mathematical representation of uncertainty. Thus, systematic analyses of the stability of optimized strategies to fluctuations in input variables need to be performed by numerical methods. Different approaches may then be viable, including Monte Carlo procedures (e.g. [73]), which will, however, require estimates of the distributions of input variables; and large-scale resampling from empirical distributions as in bootstrapping [74]. Bootstrapping has been used successfully to address uncertainty in several occupational studies addressing exposure sampling efficiency [27,53,75], and is especially useful in cases when analytical methods are unavailable [12] or when assumptions underlying the analytical models are probably violated [35,54]. Bootstrap-based analysis of uncertainty has also been used successfully in health economics [76]. However, bootstrapping requires access to - preferably large - empirical data sets that can be used to represent the distributions of necessary variables. In the case of cost-efficiency optimization, this implies that extensive data, not available at present, are needed on unit costs, exponents in the cost function, and exposure variance components.

#### Deviations from the optimal strategy

For pragmatic reasons, exposure assessments in working life will rarely be carried out as planned (e.g. [37]). Thus, an intended optimal strategy may, in effect, be realized by collecting numbers of measurement units at different stages that deviate from the optimal choice, even if the total budget is still consumed. Presumably, the most likely deviations to occur appear in the form of slight departures from a completely balanced data set; for instance that some measurement occasions fail for some subjects but are compensated by more occasions from others. As noted from the numerical examples (table 2), statistical performance seems to be considerably more sensitive to non-optimal strategies at some combinations of variance components, unit costs and cost function exponents than at others. However, this result concerns only non-optimal strategies that are still balanced. The effects of unbalanced reallocations of measurements, which still consume the allowed budget, need to be determined in future studies. When facing scenarios that will be sensitive to deviations from the optimal strategy, we suggest, however, preparing for likely departures by designing an intentional oversampling.

### Comparing cost-efficiencies

#### Comparing measurement allocations

Some previous studies on cost-efficient data collection have been devoted to comparing two or more alternative measurement strategies with respect to cost and efficiency, rather than identifying an optimal strategy. Thus, Armstrong compared the properties of two different instruments for retrieving the same exposure data [40,41], while Lemasters et al. [38] and Shukla et al. [39] devoted their studies to comparing different allocations of measurements using the same instrument. In the two latter studies, probably none of the compared strategies were optimal, but they were meant to represent feasible strategies in terms of e.g. logistics and selection constraints. The comparison approach to cost-efficiency analysis is considerably easier to deal with from a mathematical viewpoint than optimization as addressed in the present paper. A mere comparison also allows for both cost and output variance functions that cannot be addressed by analytical optimization procedures. Abstaining from optimization may thus represent a pragmatic level of analysis in cases where the principal objective is to decide for one of a number of possible exposure assessment strategies rather than determining an absolute optimum.

#### Comparing measurement instruments

While, as mentioned, some previous studies have addressed the issue of comparing the cost-efficiency of two alternative methods for obtaining the same exposure variable(s) [40,41], no attempts have been made on comparing two instruments in terms of their *optimal *performance under a constrained budget. This is an issue of obvious importance to a researcher or practitioner facing a decision on investments in new equipment or staff. For many occupational and environmental exposures, several alternative measurement instruments are available. For instance, working postures can be recorded using self-reports, observations and direct measurement tools [77,78]; i.e. methods associated with different costs and different statistical performance [79,80]. The procedures developed in the present paper can be used to identify an optimal measurement strategy for each method separately, including the resulting statistical performance, on which basis a comparison can be made. In this case, it is particularly important to acknowledge fixed costs with either method, since they determine the budget left for optimization.

## Conclusion

In the present study, we demonstrated that non-linearities in costs functions can have a significant influence on the *principle *of how to optimally allocate measurements between subjects and occasions within subjects. This happens if costs for recruiting subjects are large compared to costs for setting up measurement occasions, and, at the same time, the between-subjects to within-subject variance ratio is small. If, on the other hand, the between-subjects variance is larger than or similar to the within-subject variance, non-linearities do not, in general, change the supremacy of measuring at one occasion from each of as many subjects as allowed by the budget. This principle applies in particular if the budget is large. Irrespective of the extent of exposure variability, however, non-linear subject recruitment costs will affect the eventual *size *of the exposure data sample, and hence the precision of the resulting exposure mean value.

We noted a remarkable scarcity of empirical data on appropriate approximations of cost functions in exposure assessment, as well as on the sizes of costs pertaining to different measurement stages, for instance subjects and occasions within subjects.

Thus, in epidemiologic research requiring reliable exposure mean values, we suggest that exposure assessment strategies are discussed *a priori*, using the procedures developed in the present paper on educated estimates of relevant variance components, unit costs, and cost function shapes. This should lead to informed decisions on measurement strategies that pursue an optimal use of monetary resources, with due consideration as to whether the obtainable statistical performance is sufficient.

## Competing interests

The authors declare that they have no competing interests.

## Authors' contributions

SEM conceived of the study, derived some of the analytical procedures, performed all numerical simulations, and drafted major parts of the manuscript. KB derived most of the analytical procedures, and drafted significant parts of the manuscript. Both authors read and approved the final manuscript.

## Appendix

The conditions for the objective function to be convex if *β *≠ 1 (case B), can be derived as follows:

First, take the derivative of equation (14) with respect to *n*_*d*_:

This expression will always be positive for *β *≥ 2, and hence the objective function (equation (13)) convex. For *β *< 2 sufficient conditions for convexity follow from the inequality:

This last inequality is equivalent to:

, i.e.:

This inequality is true if *β*-1 and  are both positive or both negative.(A1)(A2)

Thus, to summarize, the objective function is always convex for *β *≥ 2. For 1 <*β <*2 and *β *< 1, it is convex if inequalities A1 and A2 apply, respectively.

## Pre-publication history

The pre-publication history for this paper can be accessed here:

http://www.biomedcentral.com/1471-2288/11/76/prepub
